# Can conservative treatment be effective for thoracolumbar injuries patients with TLICS scores of 4 or 5? An analysis of initial radiological findings and clinical risk factors for treatment failure

**DOI:** 10.1186/s12891-024-07543-6

**Published:** 2024-06-03

**Authors:** Eun Kyung Khil, Il Choi, Kyoung Yeon Lee, Young Woo Kim

**Affiliations:** 1 Department of Radiology, Hallym University Dongtan Sacred Heart Hospital, 7, Keunjaebong-gil, Hwaseong-si, 18450 Gyeonggi-do Korea; 2 Department of Radiology, Fastbone Orthopedic Hospital, 127-27, Dongtansunhwan-daero, Hwaseong-si, Gyeonggi-do Korea; 3 Department of Neurological Surgery, Hallym University Dongtan Sacred Heart Hospital, 7, Keunjaebong-gil, Hwaseong-si, 18450 Gyeonggi-do Korea; 4 Department of Orthopedic Surgery, Hallym University Dongtan Sacred Heart Hospital, 7, Keunjaebong-gil, Hwaseong-si, Gyeonggi-do 18450 Korea

**Keywords:** Thoracolumbar fracture, Spine fracture, Posterior ligamentous complex, MRI, Cobb angle, TLICS (thoracolumbar Injury classification and severity)

## Abstract

**Background:**

This study aimed to assess the outcomes of conservative management in patients with thoracolumbar fractures classified with a Thoracolumbar Injury Classification and Severity (TLICS) score of 4 or 5, and to analyze initial imaging findings and clinical risk factors associated with treatment failure.

**Methods:**

In this retrospective analysis, patients with thoracolumbar fractures and a TLICS score of 4 or 5, determined through MRI from January 2017 to December 2020, were included. Patients undergoing conservative treatment were categorized into two groups: Group 1 (treatment success) and Group 2 (treatment failure), based on initial and 6-month follow-up outcomes. Clinical data were compared between the two groups. Initial radiological assessments included three kyphosis measurements (Cobb angle, Gardner angle, and sagittal index [SI]), anterior and posterior wall height, and central canal compromise (CC). Additionally, risk factors contributing to treatment failure were analyzed.

**Results:**

The conservative treatment group comprised 84 patients (mean age, 60.25 ± 15.53; range 22–85; 42 men), with 57 in Group 1 and 27 in Group 2. Group 2 exhibited a higher proportion of women, older age, and lower bone mass density (*p* = 0.001–0.005). Initial imaging findings in Group 2 revealed significantly greater values for Cobb angle, SI, and CC (*p* = 0.001–0.045 or < 0.001; with cutoff values of 18.2, 12.8, and 7.8%, respectively), and lower anterior wall height (*p* = 0.001), demonstrating good to excellent interobserver agreement (0.72–0.99, *p* < 0.001). Furthermore, osteoporosis was identified as a significant risk factor (odds ratio = 5.64, *p* = 0.008).

**Conclusion:**

Among patients with TLICS scores of 4 or 5, those experiencing conservative treatment failure exhibited unfavorable initial radiological findings, a higher proportion of women, advanced age, and osteoporosis. Additionally, osteoporosis emerged as a significant risk factor for treatment failure.

## Background

Since the Thoracolumbar Injury Classification and Severity (TLICS) system was first introduced in 2005 [1], suggestions for validation and modification have been made [2, 3]. Since then, the TLICS system has consistently proven reliable in the clinical field. The TLICS provides a treatment guide for thoracolumbar (TL) fractures using a scoring system. However, as the TLICS system was established using the Delphi method of inquiry by international experts in the clinical field, it is challenging to apply the same standard to all patients [1].

When the TLICS system was created in 2005, the scoring system was used even for patients without MRI data. Currently, TLICS is generally assessed through MRI [4–6]. Because MRI has high sensitivity for detecting fractures and is used as the gold standard for evaluating posterior ligamentous complex (PLC) injuries, MRI is performed for most fracture evaluations [4, 7–9]. Consequently, the reliance on radiologists has increased in terms of TLICS scoring via MRI.

A score of 4 or higher in the TLICS system is typically considered for surgery [10, 11]. However, in the clinical field, there are many cases in which not all patients with a score of 4 or higher require surgery. This is common in TLICSs 4 and 5. Previous studies on nonsurgical patients have included patients with a score of 4 or less [12]. Since 4 points depend on the physician’s opinion, previous studies have focused on factors that determine surgery efficacy. Unlike these studies, we included the TLICS 5 group and hypothesized that ‘not all TLICS patients with scores of 4 or 5 require surgery’. Although surgeries offer distinct advantages, their potential complications, such as infections and instrument failures, could result in costs for the healthcare system.

We retrospectively observed patients with TLICS scores of 4 or 5 who did not undergo surgery and investigated imaging and clinical risk factors for the treatment failure group. The purpose of our study was to investigate the morphological changes in the spinal column over time in patients with TLICS 4 or 5 who received conservative treatment and to analyze the risk factors for treatment failure; these patients had significant kyphotic changes and may have required additional treatment, such as pain procedures or instrument fixation.

## Methods

### Patient selection

This retrospective study was approved by the Institutional Review Board, and informed consent was not needed. From January 2017 to January 2021, adult patients who underwent thoracic or lumbar spine MRI in the picture archiving and communication system (PACS) were enrolled if they had an acute TL fracture less than one month old. A total of 863 patients were enrolled.

**Fig. 1:**
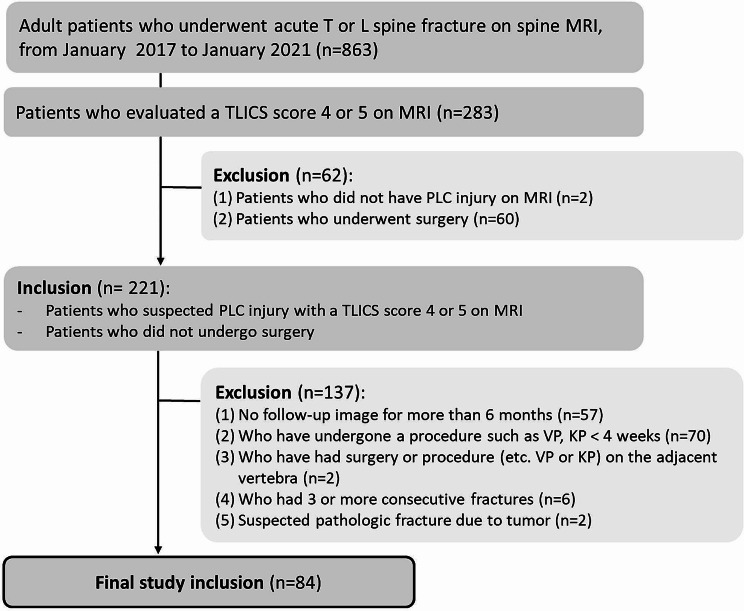
Flowchart of study patient inclusion (TLICS = thoracolumbar injury classification and severity, PLC = posterior ligamentous complex, VP = vertebroplasty, KP = kyphoplasty)

The inclusion criterion was patients suspected of having PLC injury with a TLICS score of 4 or 5 on MRI who did not undergo surgery opt for surgery based on the individual philosophy of their surgeons. There were 283 patients with TLICSs of 4 or 5 (136 patients with 4 points and 87 patients with 5 points, respectively). Of these, two patients did not have PLC injury, and 60 patients underwent surgery. Thus, 221 patients were included. The exclusion criteria were as follows: did not have a follow-up image for more than 6 months (*n* = 57), had undergone a procedure such as vertebroplasty or kyphoplasty at the fracture site within 4 weeks (*n* = 70), had surgery or a procedure (e.g., vertebroplasty or kyphoplasty) on the adjacent vertebra (*n* = 2), had 3 or more consecutive fractures (*n* = 6), or had a suspected pathologic fracture due to a tumor (*n* = 2). A total of 84 patients were included in this study after excluding 137 patients (Fig. 1).

### Initial evaluation of MRI data with TLICS scoring and severity of back pain

For the evaluation of TL fractures, 3.0T MRI (Skyra, Siemens Healthineers) was used. The spinal MRI protocol included axial and sagittal T2- and T1-weighted images and sagittal T2-weighted fat saturation images. MRI images of all 863 patients with acute TL fractures were evaluated by TLICS scoring by a single radiologist (a musculoskeletal radiologist with 10 years of experience; reader 1). The TLICS is classified according to fracture morphology (0–4 points), PLC injury (0–3 points), and neurological symptoms (0–3 points), and the total score is expressed as the sum of these scores [1, 13]. In most cases of fracture morphology, compression or burst fracture was evaluated as 1 or 2 points, respectively, and those in which the PLC was not intact were evaluated as 2 or 3 points (possible PLC injury 2 points, injured PLC 3 points). Since this study included patients with suspected PLC injury (PLC 2 or 3), only those patients with a TLICS score of 4 or 5 were included only because of the sum of the fracture morphology and PLC injury. Therefore, these patients had posterior column injuries without neurological deficits. Back pain intensity was measured using the visual analog scale (VAS) on a scale of 0–10; pain intensity is routinely measured on the day the patient visits the hospital and the patient’s medical records are available.

### Imaging analysis of vertebral body fractures

To assess kyphotic changes considered indicators of treatment failure in patients with spinal fracture, the three most widely utilized methods for measuring kyphotic angles were employed. Additionally, representative metrics for evaluating the severity of the fracture and the potential for treatment failure, namely, the compression rate of vertebral body height (CVBH) and central canal compromise (CC) rate, were utilized. For image analysis, we used images from the initial hospital visit due to the fracture. For KA and CVBH, static images of the L-spine or T-spine lateral radiography were used. For the CC rate, sagittal computed tomography (CT) images were used. When CT was not performed, the T2-weighted image was measured via sagittal T2-weighted imaging. Image analyses were performed by two radiologists (reader 1 and reader 2 [second year of radiology resident]) blinded to each other’s measurement results. Interobserver agreement between the two readers was obtained.

**Fig. 2:**
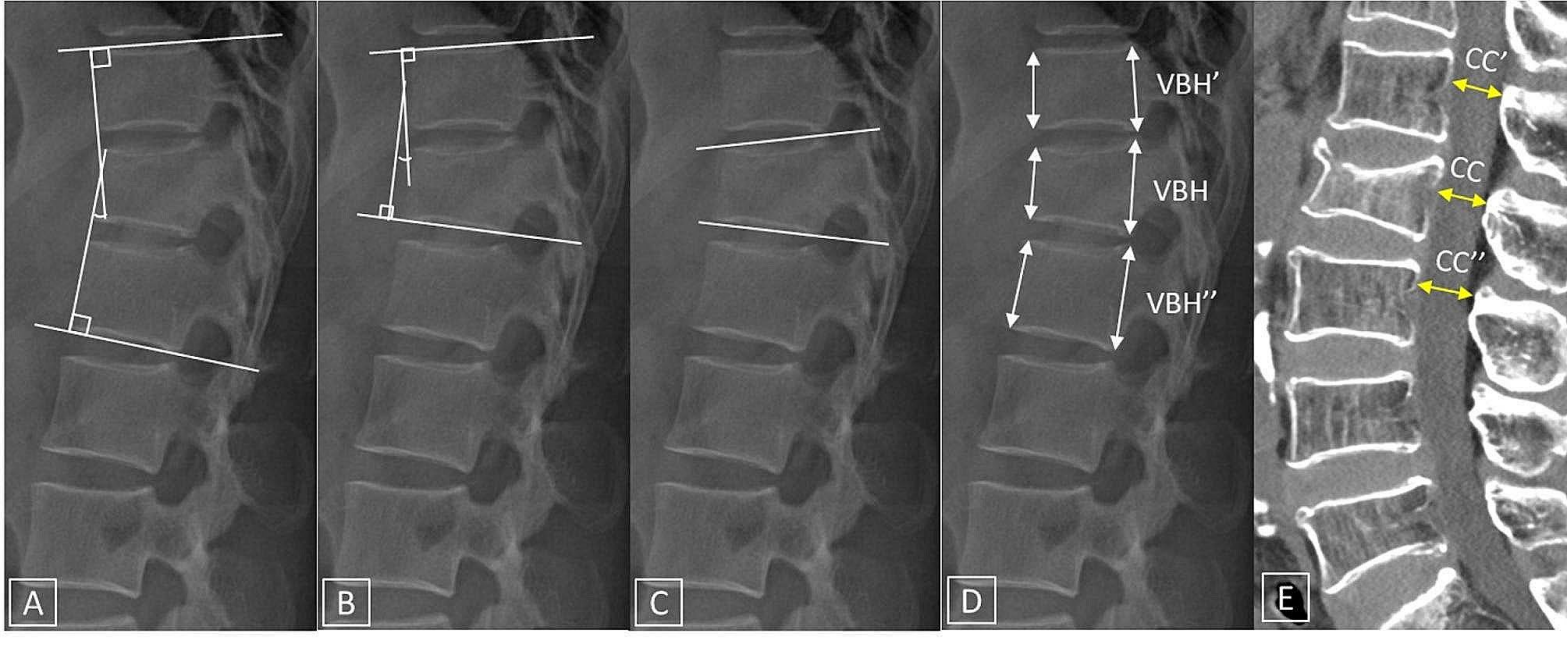
Fracture-related imaging measurements: A-C, KA measurements (A, Cobb angle; B, Gardner angle; C, sagittal index); D, CVBH measurement (anterior wall [dashed arrow], posterior wall [arrow]); E, CC rate measurement. D, formula for CVBH: CVBH (%) = VBH/[(VBH’+VBH”)/2]x100%. E, Formula for CC rate: CC rate (%) = 1-[2×CC/(CC’+ CC”)×100%] (KA = kyphotic angle, VBH = vertebral body height, CVBH = compression rate of vertebral body height, CC = central canal compromise)

KA was measured according to three methods (Fig. 2a-c): the Cobb angle (CA, KA-1), the Gardner angle (KA-2), and the sagittal index (SI, KA-3). The CA is a measure of the angle between the line connecting the lower endplate of the vertebral body one level below the fractured vertebral body and the line connecting the boundary of the upper endplate of the vertebral body one level above the fractured vertebral body. The Gardner angle is a measure of the angle between the fractured vertebral body and the body above it, and the SI is a measure of the angle of the line connecting the boundary of the upper and lower endplates of the fractured vertebral body. A positive value was indicated in the presence of kyphosis, and a negative value was indicated in the presence of lordosis.

CVBH was measured at both the anterior and posterior walls of the vertebral body (Fig. 2d). The lengths of the anterior and posterior walls of three consecutive vertebral bodies, including the fracture site, were measured, and the compression ratio of the height of the fractured vertebral body was calculated by calculating the average height of the upper and lower vertebrae.

To measure the CC rate (Fig. 2e), the diameter of the narrowest central canal of the fractured vertebral body was measured via sagittal CT or MR scan, and the average diameter of the central canal measured at the upper and lower vertebrae of the fractured vertebral body was obtained. Then, the compromise rate of the fracture site was calculated.

### Treatment outcome of fracture

**Fig. 3:**
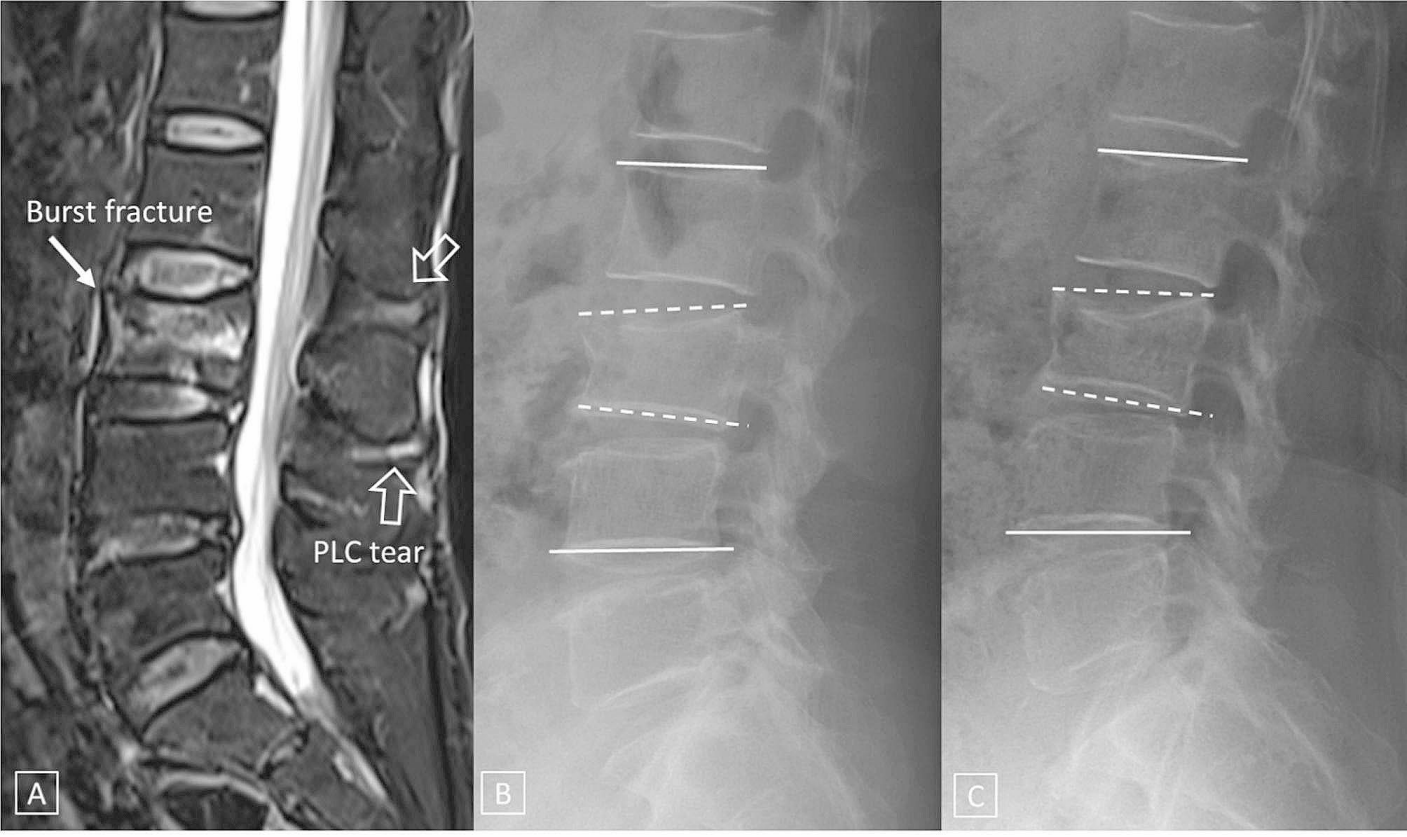
A 71-year-old woman with a TLICS 5 score and treatment success. A, On MRI with a T2-weighted fat saturation sagittal scan, an acute burst fracture was observed at the L3 vertebral body (arrow), and a PLC tear (open arrows) was observed at the L2/3 and L3/4 segments. Initial radiography (B) and radiography (C) after 6 months showed no significant changes in KAs. (initial measurements: Cobb angle= -1.1, Gardner angle = 6.24, sagittal index = 8.1; 6 months later: Cobb angle= -1.8, Gardner angle = 7.1, sagittal index = 8.52) (TLICS = thoracolumbar injury classification and severity, PLC = posterior ligamentous complex, KA = kyphotic angle)

**Fig. 4:**
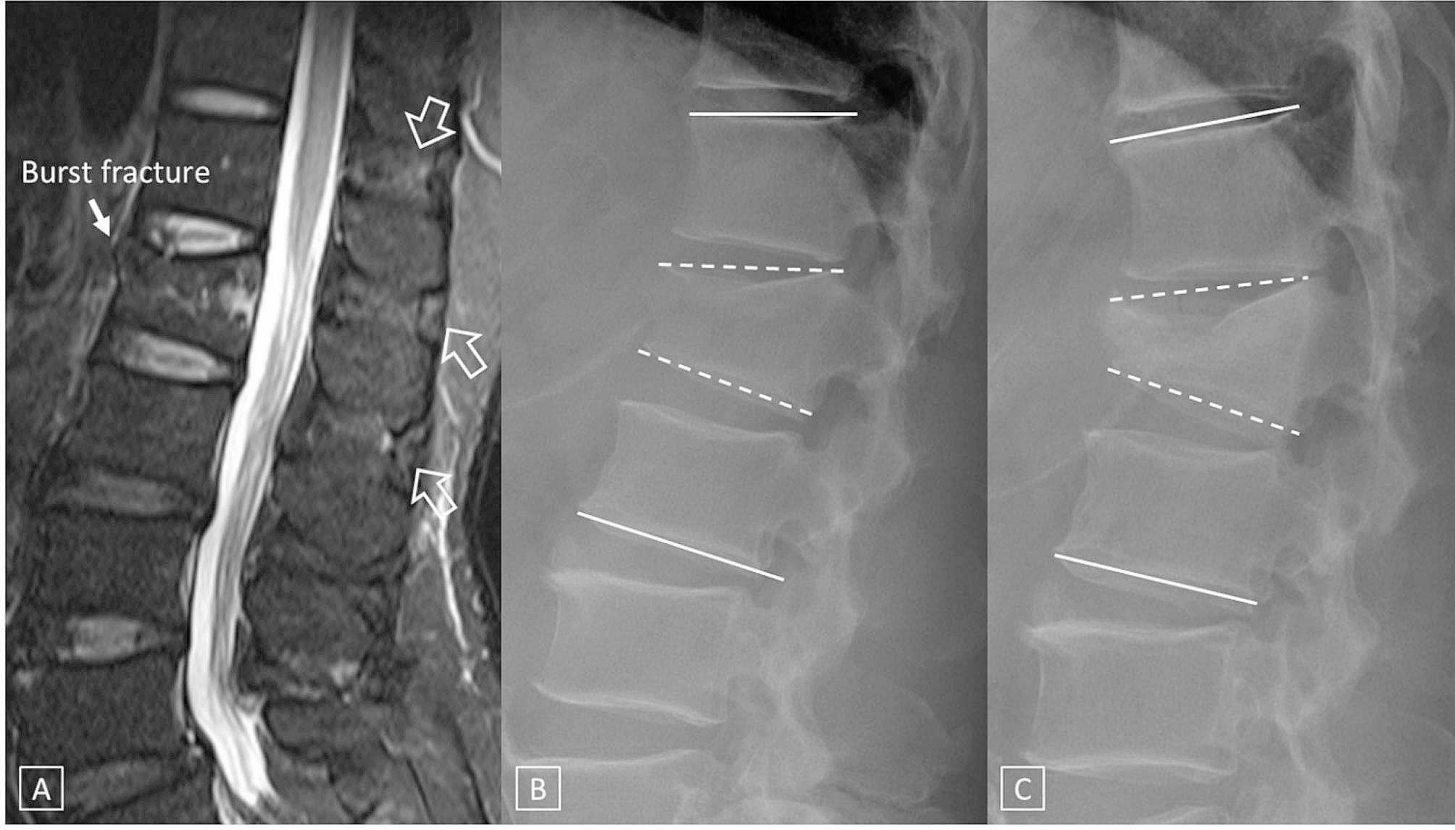
A 62-year-old man with a TLICS score of 4 and treatment failure. A, On a sagittal MRI scan, an acute burst fracture was observed at the L2 vertebral body (arrow), with a possible PLC tear (open arrows) at the T12/L1, L1/2, and L2/3 segments. Initial radiography (A) and radiography (B) after 6 months showed significantly increased kyphotic angles. (initial measurements: Cobb angle = 8.1, Gardner angle = 11.1, sagittal index = 13.0; 6 months later: Cobb angle = 24.7, Gardner angle = 30.5, sagittal index = 24.9) (TLICS = thoracolumbar injury classification and severity, PLC = posterior ligamentous complex)

Patients who underwent initial conservative treatment were divided into two groups based on their initial and 6-month follow-up outcomes: group 1 (treatment success) and group 2 (treatment failure). Initial conservative management included two weeks of prescribed bed rest, utilization of a Thoracolumbosacral Orthosis (TLSO) brace for spinal stabilization, administration of analgesic medication for pain relief, and commencement of osteoporosis treatment upon confirmation. Treatment failure was defined as conversion to surgery or an interventional procedure such as delayed vertebroplasty or kyphoplasty due to intolerable pain at least one month of conservative treatment or progressive kyphosis for more than 6 months. Progressive kyphosis was defined as a case in which the CA increased by more than 10 degrees on radiography performed after 6 months, according to the definition of junctional failure of the existing proximal junction kyphosis [14, 15] (Figs. 3 and 4).

### Risk factor analyses for treatment failure

The following risk factors were investigated in patients with treatment failure (group 2): age, sex, body mass index (BMI), height, weight, bone mass density (BMD), kyphotic angles (KA-1, 2, 3), CVBH (anterior and posterior wall), and CC rate. BMI was analyzed by dividing it into two categories based on 25 variables, which indicate overweight. BMD was divided into two categories based on the presence of osteoporosis, defined by a T score of -2.5 or less.

### Statistical analyses

For comparative analysis between groups 1 and 2, the chi-square test, t test or Mann‒Whitney test was used for demographic and fracture-related variables. The kyphotic angle, CVBH, and CC discordance rate obtained from the radiographic imaging data were also comparatively analyzed via t tests. The receiver operating characteristic (ROC) curve and area under the curve (AUC) were obtained for these three types of radiographic imaging data for the treatment failure group (group 2), and the cutoff value was determined. Interobserver agreement between the two reviewers for radiographic imaging data was analyzed using the intraclass correlation coefficient (ICC).

To analyze the significant variables among the risk factors for treatment failure, univariate logistic regression analysis was performed, and multivariate logistic regression analysis for predicting treatment failure was performed using the predictive variables.

All the statistical analyses were conducted using SPSS Statistics 20.0 and SAS software 9.4.

## Results

A total of 84 patients were included in the conservative treatment group (mean age, 60.25 ± 15.53; range 22–85; 41 men), with 57 (67.86%) in group 1 and 27 (32.14%) in group 2 (Table 1). Surgical fixation was recommended for a minor subset of these 27 patients (4.76%, 4/84). The reasons for this surgical intervention were pain or functional impairment (1 patient), neurologic deterioration (1 patient), and worsening kyphotic deformity (2 patients). Concurrently, delayed osteoplasty (> 1 month) was performed in another 12 patients (14.29%, 12/84) to alleviate pain. Furthermore, an increase in kyphotic angulation, measuring at least 10 degrees, was observed in 11 patients (13.09%, 11/84).

**Table 1:** Characteristics of patients with nonoperative thoracolumbar fractures

	Total (*n* = 84)	Group 1 (*n* = 57)	Group 2(*n* = 27)	*P* value	
Sex					
Men	41 (48.8%)	34 (59.6%)	7 (25.9%)	0.004*	
Women	43 (51.2%)	23 (40.4%)	20 (74.1%)		
Age (years)	60.25 [15.53]	56.23 [15.77]	68.74 [11.13]	< 0.001*	
Height (cm)	162.50 [10.86]	165.28 [10.39]	156.63 [9.54]	< 0.001*	
Weight (kg)	63.98 [13.90]	65.86 [14.64]	60.00 [11.43]	0.071	
BMI (m^2^/kg)	24.08 [3.79]	23.93 [3.95]	24.38 [3.50]	0.612	
BMD (T score)	-1.95 [1.47]	-1.52 [1.38]	-2.71 [1.34]	0.001*	
Pain to initial study interval (day)	3.78 [9.80]	3.23 [8.47]	4.78 [12.10]	0.502	
VAS, initial	5.33 [2.56]	5.35 [2.64]	5.30 [2.43]	0.928	
TLICS score					
4	49 (58.3%)	32 (56.1%)	17 (63.0%)	0.554	
5	35 (41.7%)	25 (43.9%)	10 (37.0%)		
Fracture morphology					
1(compression)	6 (7.1%)	4 (7.0%)	2 (7.4%)	0.948	
2(burst)	78 (92.9%)	53 (93.0%)	24 (92.6%)		
PLC injury					
2(possible)	43 (51.2%)	28 (49.1%)	15 (55.6%)	0.582	
3(tear)	41 (48.8%)	29 (50.9%)	12 (44.4%)		
Main fracture level					
T1-9	8 (9.5%)	6 (10.5%)	2 (7.4%)	0.581	
T10-L2	59 (70.2%)	38 (66.7%)	21 (77.8%)		
L3-5	17 (20.2%)	13 (22.8%)	4 (14.8%)		

† BMI = body mass index, BMD = bone mass density, VAS = visual analog scale, TLICS = thoracolumbar injury classification and severity, PLC = posterior ligamentous complex

‡ [ ]: standard deviation; * *p* < 0.05

### Comparison of demographic and fracture characteristics between the two groups

In group 2, there were significantly more women (74.1%, *p* = 0.004), and the mean age was significantly greater (68.74 vs. 56.23 in group 1, *p* < 0.001) (Table 1). The average T score of Group 2 was − 2.71, which was significantly different from the T score of Group 1 (*p* = 0.001). However, BMI, pain duration, and initial VAS score did not significantly differ between the two groups. All of the patients had compression or burst type TL fractures. For the variables related to fracture, there was no significant difference between the two groups in terms of the TLICS score, which are indicators of fracture severity, the degree of PLC injury, or the main fracture level.

### Comparison of initial fracture imaging findings between the two groups

For the kyphotic angle (KA-1, KA-2, or KA-3), the values in group 2 were 11.53, 13.96, and 14.38, respectively, which were greater than those in group 1. Of these, KA-1 and KA-3 had statistically significant differences (*p* = 0.042 and 0.001, respectively) (Table 2). In the CVBH cohort, the height at the anterior wall was significantly lower (72.37%) (*p* = 0.001), and the degree of CC rate was higher in group 2 (*p* < 0.001). The ICCs (and 95% confidence intervals [CIs]) of the interobserver agreement between the two readers were as follows: 0.992 (0.988–0.99), 0.986 (0.979–0.991), and 0.966 (0.948–0.978) for KA-1, KA-2, and KA-3, respectively; for CVBH, the anterior wall was 0.950 (0.924–0.96), the posterior wall was 0.788 (0.691–0.857), and the CC rate was 0.726 (0.607–0.813) (all p values < 0.001).

**Table 2:** Comparison of initial radiographic findings between group 1 and group 2

	Total; *n* = 84	Group 1; *n* = 57	Group 2; *n* = 27	*P* value
Kyphosis (angle)				
- Kyphosis 1	6.99 (14.17)	4.84 (14.93)	11.53 (11.39)	0.042*
- Kyphosis 2	10.89 (10.04)	9.43 (10.17)	13.96 (9.19)	0.053
- Kyphosis 3	11.17 (6.24)	9.65 (6.09)	14.38 (5.38)	0.001*
CVBH (%)				
- Anterior wall	78.74 (12.30)	81.75 (10.75)	72.37 (13.10)	0.001*
- Posterior wall	95.14 (5.71)	94.81 (6.21)	95.84 (4.51)	0.446
Canal compromise rate (%)	10.01 (10.08)	7.34 (9.54)	15.67 (8.94)	< 0.001*

† CVBH = compression rate of vertebral body height

‡ (): standard deviation; * *p* < 0.05

### Diagnostic performance of radiographic parameters for treatment failure

When the diagnostic performance for treatment failure was analyzed, the KA-3 level was 0.72, and the cutoff value was 12.96, for which the sensitivity and specificity were 62.96% and 70.18%, respectively (Table 3). For CVBH, the AUC of the anterior wall was 0.70, the cutoff value was 79.47%, and the sensitivity and specificity were 62.96% and 71.93%, respectively. The AUC of the CC was 0.76, which was the highest among all the radiological variables, and the cutoff was 8.00%, with a sensitivity of 81.48% and specificity of 57.90.

**Table 3:** Diagnostic performance and cutoff values for diagnosing group 2

	Cutoff value	Sensitivity	Specificity	AUC
Kyphosis (angle)				
- Kyphosis 1	18.33	40.74%	82.46%	0.63
- Kyphosis 2	8.82	85.19%	38.60%	0.64
- Kyphosis 3	12.96	62.96%	70.18%	0.72*
CVBH (%)				
- Anterior wall	79.47	62.96%	71.93%	0.70*
- Posterior wall	96.55	51.85%	63.16%	0.53
Canal compromise rate (%)	8.00	81.48%	57.90%	0.76*

† CVBH = compression rate of vertebral body height

### Risk factors for treatment failure

Among the factors for treatment failure, the statistically significant risk factors identified through univariate logistic regression analysis were female (odds ratio [OR] = 3.23, *p* = 0.027), age 60 or older (OR = 8.44, *p* = 0.007), and a BMD (T score) less than − 2.5, which are indicative of osteoporosis (OR = 9.09, *p* < 0.001) (Table 4). The significant radiological variables included KA-3 (OR = 1.16, *p* = 0.006), anterior CVBH (OR = 0.94, *p* = 0.006), and the CC rate (OR = 1.08, *p* = 0.003). After multivariable logistic regression analysis of Model 1 (BMD, KA-1, KA3, anterior CVBH, and CC rate) was performed, only osteoporosis (OR = 13.15, *p* = 0.002) was found to be significantly different. When multivariate logistic regression analysis was performed with Model 2 (age ≥ 60 years, BMD, KA3), both osteoporosis (OR = 5.88, *p* = 0.008) and KA-3 (OR = 1.14, *p* = 0.018) were significantly different.

**Table 4:** Risk factors for treatment failure

	Univariable			Multivariable (model 1)			Multivariable (model 2)		
Factors	OR	95% CI	*P value*	OR	95% CI	*P value*	OR	95% CI	*P value*
Sex									
Men	1								
Women	3.23	1.14–9.17	0.027*						
Age	1.07	1.02–1.12	0.006*						
<60	1						1		
≥60	8.44	1.78–40.08	0.007*				3.91	0.72–21.3	*0.115*
BMI (kg/m2)	1.01	0.89–1.14	*0.933*						
≤25	1								
>25	1.14	0.43–3.03	*0.792*						
Height (cm)	0.93	0.88–0.98	0.007*						
Weight (kg)	0.97	0.93-1.00	*0.088*						
BMD (T score)	0.50	0.32–0.79	< 0.001*						
> -2.5	1								
≤ -2.5	9.09	0.03–0.35	< 0.001*	13.15	2.53–68.48	0.002*	5.88	0.04–0.63	0.008*
Kyphosis (angle)									
- Kyphosis 1	1.04	0.99–1.08	*0.093*	1.03	0.96–1.11	*0.407*			
- Kyphosis 2	1.05	0.99–1.12	*0.092*						
- Kyphosis 3	1.16	1.04–1.28	0.006*	1.15	0.94–1.13	*0.174*	1.14	1.01–1.27	0.018*
CVBH (%)									
- Anterior wall	0.94	0.90–0.98	0.006*	1.04	0.96–1.13	*0.300*			
- Posterior wall	1.04	0.95–1.14	*0.356*						
CC rate (%)	1.08	1.03–1.14	0.003*	1.06	0.99–1.13	*0.097*			

† OR = odds ratio, CI = confidence interval, BMI = body mass index, BMD = bone mass density, CVBH = compression rate of vertebral body height, CC = central canal compromise

‡ * *p* < 0.05

## Discussion

The main finding of this study is that among patients with TLICS scores of 4 or 5 who received conservative treatment, approximately one-third (32.14%) had significant changes in kyphotic alignment or required additional procedures or surgery. Among these patients, only a small number (4 patients, 4.76%) ultimately required surgical fixation.

Surgical intervention was recommended according to the surgeon’s philosophy for patients with a TLICS score of 4 and for patients with a TLICS score of 5 [1]. However, this was not the case in our study.

The reason why surgery is not required for TLICS grade 4 or 5 patients can be considered as follows. When the TLICS was released, MRI was not popularized before 2005 as a reference data. Radiography, CT, and MRI were combined to evaluate PLC injury, making it difficult to consistently and clearly evaluate PLC injury [1]. Since then, MRI has become common, and PLC injuries, even those with relatively fewer injuries, have been accurately and sensitively evaluated [13, 16]. As a result, even the group with less severe PLC injury than that evaluated by conventional radiography or CT was also included if their TLICS score was 4 or 5. Moreover, conservative treatment is not inferior in patients classified as having stable bursting fractures according to previous studies [11]. The patient group included in the above study was evaluated for bursting fracture by only radiography or CT, and it was not clear whether the PLC was injured. Therefore, at present, when MRI is a common and standard imaging modality, surgery may not be necessary for all patients with TLICS scores of 4 or 5.

Surgical treatment is accompanied by various complications following surgery, such as infection and instrument failure, and the cost of using implants is high [11]. If a group that can be treated with nonsurgical treatment is identified, additional costs and complications associated with surgical treatment can be avoided. In our investigation, a markedly low proportion of patients necessitated surgical fixation during the six-month follow-up period with conservative treatment. This rate stands in stark contrast to findings from previous studies. The literature reports a significantly greater rate of surgical fixation requirements during the follow-up period among patients initially treated conservatively with a TLICS score of 3 or less. Specifically, previous studies have reported rates of 19.3% (25 out of 129 patients) and 23.8% (16 out of 67 patients) [10, 12].

As established from previous research, the role of surgical treatment in preventing neurological injury and reducing the progression of kyphotic angulation in patients with TLICS scores of 4 or 5 is significant, allowing patients to return to their normal work and daily activities more quickly. However, our findings contribute to the possibility of conservative treatment being effective for patients with TLICS scores of 4 or 5. Although our exclusion of patients initially deemed to require surgery may have influenced the outcome, our results nevertheless suggest that conservative treatment can indeed be a viable option for carefully selected patients, even those presenting with higher TLICS scores of 4 or 5. This underlines the importance of meticulous patient selection in the determination of treatment pathways.

Our findings indicated that radiologic parameters reflecting greater deformities resulting from trauma, such as larger CAs, larger SIs, and greater CC rates in the initial images, were identified as significant risk factors for treatment failure. These observations align with previous studies reporting female, older age, higher initial VAS score, and wider interpedicular distance as factors associated with poor outcomes in conservative treatment [10, 12]. In our study, the average CA in the treatment failure group was 11.53°, which was lower than the previously reported CA of 18.06° in patients who required surgery [17]. Additionally, the percentage of anterior CVBH in patients requiring surgical intervention was 72.37% (± 13.10, *p* < 0.001), whereas it was 63.51% in the nonsurgery group [12] and 71.27% in another study [17]. Another notable strength of our study was the determination of cutoff values for radiological factors that can predict treatment failure, along with the confirmation of their diagnostic performance. Specifically, we identified a significant cutoff value of 12.96° for the SI (AUC = 0.76) and 8.0% for the CC (AUC = 0.76). Furthermore, a significant decrease in anterior CVBH was observed, with a cutoff value of 79.47% (AUC = 0.70). This aspect of our study holds particular significance because it represents the first attempt to determine the diagnostic performance of these radiological factors using AUC analysis and establish cutoff values for their predictive utility.

In our study, osteoporosis was a single significant risk factor in the group of patients who experienced treatment failure with conservative management. The stability of the spine can be considered to be affected, and the stability may not be secured. In particular, since the anterior column contains more cancellous bone than does the posterior column, the former progresses to a wedging shape, and the latter is considered to have belonged to the 10 degree or greater kyphosis group, which was the treatment failure group in our study. In addition, osteoporosis was included as an indication suitable for pain procedures, including vertebroplasty, if pain persists over time.

The limitations of our paper are as follows. First, our study included demographic and radiological factors but did not address the VAS or Oswestry Disability Index scores, which are associated with clinical outcomes. Second, the degree of kyphosis defined as treatment failure varies from 10 to 20 degrees according to the literature, and depending on the degree of kyphosis, the results may be different [18]. Third, this was a retrospective study, and the sample size was small. Fourth, our study predominantly included elderly patients with osteoporosis, who are more susceptible to injuries from low-energy trauma. The outcomes might vary if the focus is solely on younger patients exposed to high-energy trauma. Finally, our follow-up period might be insufficient to fully capture the long-term effectiveness of treatment outcomes; a longer follow-up duration could provide more comprehensive insights.

To conclude, our findings suggest that a conservative approach can be appropriate for patients with TLICS scores of 4 or 5. However, there are heightened risks of treatment inadequacies for patients with elevated CA, SI, or CC or for those diagnosed with osteoporosis. These findings could guide clinicians toward more personalized treatment decisions, aiming to enhance both the effectiveness and quality of patient care.

## Data Availability

All data generated or analysed during this study are included in this published article.

## Abbreviations

TLICS: Thoracolumbar Injury Classification and Severity
TL: Thoracolumbar
PLC: Posterior ligamentous complex
MRI: Magnetic resonance imaging
KA: Kyphotic angle
CVBH: Compression rate of vertebral body height
CA: Cobb angle
SI: Sagittal index
CC: Central canal compromise
CT: Computed tomography
VAS: Visual analog scale
BMI: Body mass index
BMD: Bone mass density
